# Long-term comparative analysis of no evidence of disease activity (NEDA-3) status between multiple sclerosis patients treated with natalizumab and fingolimod for up to 4 years

**DOI:** 10.1007/s10072-021-05127-z

**Published:** 2021-03-06

**Authors:** Tommaso Guerra, Francesca Caputo, Bianca Orlando, Damiano Paolicelli, Maria Trojano, Pietro Iaffaldano

**Affiliations:** Department of Basic Medical Sciences, Neurosciences and Sense Organs, University of Bari “Aldo Moro”, Piazza G. Cesare 11, 70124 Bari, Italy

**Keywords:** Multiple sclerosis, NEDA-3, Natalizumab, Fingolimod, Comparative analysis

## Abstract

**Background:**

Comparative effectiveness of natalizumab and fingolimod over a follow-up longer than 2 years has been not addressed yet.

**Objectives:**

To compare the effect on no evidence of disease activity (NEDA-3) in relapsing-remitting multiple sclerosis (RRMS) patients treated with natalizumab or fingolimod for at least 4 years.

**Methods:**

We included RRMS patients switched from first-line agents to natalizumab or fingolimod. Patients were propensity score (PS)-matched on a 1-to-1 basis. Percentages of patients reaching NEDA-3 status at 2 and 4 years of follow-up were compared using the chi-square test. The risk of not achieving NEDA-3 at 4 years was explored in matched samples by Cox regression models.

**Results:**

We evaluated 174 PS-matched patients. Patients receiving natalizumab reached a NEDA-3 status at 2 and 4 years more frequently than those exposed to fingolimod (63% vs 44%, *p*=0.037; 45.7% vs 25.8%, *p*=0.015, respectively). Patients receiving natalizumab were at a significant lower risk of not achieving the NEDA-3 status at 4 years compared to those exposed to fingolimod (hazard ratio (95% confidence interval): 0.54 (0.36–0.80), *p*=0.002).

**Conclusions:**

Although both medications were effective in patients non-responding to first-line agents, natalizumab seems to be superior to fingolimod in RRMS in obtaining NEDA-3 status at 4 years.

## Introduction

First-line disease-modifying therapies (DMTs), both injectables (interferon beta-1b, interferon beta-1a, glatiramer acetate) and orals (teriflunomide and dimethyl-fumarate) significantly reduce relapse rate and short-term disability worsening in multiple sclerosis (MS) [1–6].

In spite of this, a considerable number of patients treated with first-line agents continue to experience disease activity, which is associated with accumulation of disability [7, 8]. Based on the evidence from randomized clinical trials (RCTs), escalation to second-line DMTs (natalizumab (NTZ) or fingolimod (FIN)) after treatment failure of first-line BRACE (Betaferon®, Betaseron®, Rebif®, Avonex®, Copaxone®, or Extavia®), treatment therapies are more effective than the so-called lateral switch in reducing clinical and MRI disease activity [9]. This approach has been based on the perceived superior efficacy of these drugs in comparison to the injectable first-line agents [10, 11].

Since 2015 several observational studies have evaluated the comparative effectiveness of NAT vs FIN in RRMS patients non-responders to first-line therapy [12–23].

Almost all of these studies have shown the superiority of NTZ compared to FIN mainly in reducing clinical and radiological measures of inflammatory disease activity [12–23]. Most of these studies have a limited follow-up, in some cases up to 24 months, thus limiting the possibility to detect the effect on measures of disability accrual. Whether these two strategies might have a different impact on medium term disability accumulation is still to be elucidated.

Moreover, in the continually evolving topic of which is the better outcome measure to evaluate the treatment response in MS, NEDA has been proposed as a promising tool. It is based on absence of relapses, absence of sustained disability worsening, and absence of radiological activity (NEDA-3) [24, 25].

Here we report the results of an observational head-to-head analysis aimed to investigate the comparative effectiveness of NTZ or FIN on the composite score NEDA at 2 and at 4 years of follow-up in a real-life setting.

## Materials and methods

### Data collection and study population

All the data about MS history, demographics, treatments, and regular follow-up of the MS patients followed at the Multiple Sclerosis Center of the University Hospital Policlinico of Bari are collected, according to the Italian MS Registry study [26], which was approved by the ethical committee at the “Azienda Ospedaliero–Universitaria–Policlinico of Bari” using the iMed software.

In March 2018, we extracted data of patients of RRMS patients who had switched DMT from interferon beta or glatiramer acetate to either NTZ or FIN after at least one on-treatment relapse documented in the year before treatment switch and who have been continuously exposed to either NTZ or FIN for at least 4 years. In addition, we excluded patients who have been treated with NTZ before FIN and vice versa. NTZ was licensed in Italy in 2007, whereas FIN became available in Italy in 2011. Therefore, to ensure that all patients and physicians had the possibility to choose between NTZ and FIN, we restricted the analysis to patients who switched DMT after 2011.

### Study endpoints

The study endpoints were the achievement of a NEDA-3 status at 2 and at 4 years of follow-up. NEDA-3 was defined as follows: no relapses, no confirmed EDSS (Expanded Disability Status Scale) progression from baseline to second and fourth year, and no new T2 and/or gadolinium enhancement (Gd+) lesions. A relapse was defined as any new neurological symptom, not associated with fever or infection, lasting for at least 24 h and characterized by new neurological signs. We calculated for each year of follow up the annualized relapse rate (ARR), defined as the total number of relapses divided by the total person-time at risk of relapse.

Disability worsening was defined as 1.5-point increase (if baseline EDSS score was 0), 1.0-point increase (if baseline EDSS score was < 5.5), or 0.5-point increase (if baseline EDSS score was 5.5) confirmed 6 months apart.

Radiological activity was defined as the occurrence of Gd-enhancing lesion or new/enlarged T2-hyperintense lesions. Both brain MRI and MRI of the spinal cord were included in the assessment of NEDA, and Gd enhancement was performed at all scans collected. MRI assessment was performed approximately once a year.

The NEDA status was considered reached only if all the above parameters were fulfilled.

### Matching and statistical analysis

In order to reduce the impact of the selection and of the indication biases, the patients included in this study were matched on their propensity for receiving NTZ. The propensity score (PS) was based on a multivariable logistic regression model with treatment allocation as the dependent variable and the demographic and clinical variables available to treating neurologists at the time of the treatment decision as the independent variables.

The following covariates were included in the model: age, sex, disease duration, total number of relapses, and the number of relapses in the year prior to the treatment switch, previous cumulative DMT exposure, EDSS score, washout time from first-line DMT to FIN or NTZ, comorbidity (yes/no), and number of new MRI T2 and of Gd+ lesions. Then patients were matched in a 1:1 ratio using nearest neighbor matching within a caliper of 0.1 standard deviations of the PS.

The quality of the match in each pair of matched cohorts was assessed with standardized mean difference (SMD). SMD less than 10% was considered acceptable. The adequacy of the matching has been also validated through graphic methods, deriving from the elaboration of the PS matching (PS graphs). Summaries of continuous variable have been calculated as median with interquartile ranges (IQR) or mean and standard deviation (SD); categorical variables have been presented as frequencies (proportions).

Between-group comparisons were performed by using the Mann-Whitney test (for continuous variables) or the chi-square test (for categorical variables). Percentages of patients reaching NEDA-3 status at 2 and 4 years of follow-up were compared using the chi-square test. The hazard (along with the 95% confidence interval (CI)) of not achieving NEDA-3 status at 4 years was explored in matched samples by Cox proportional hazard regression models, adjusted for PS covariates and stratified by matched cases. All assumptions for Cox regression model were fulfilled. All analyses were performed with SPSS software version 22.0.

## Results

**Fig. 1:**
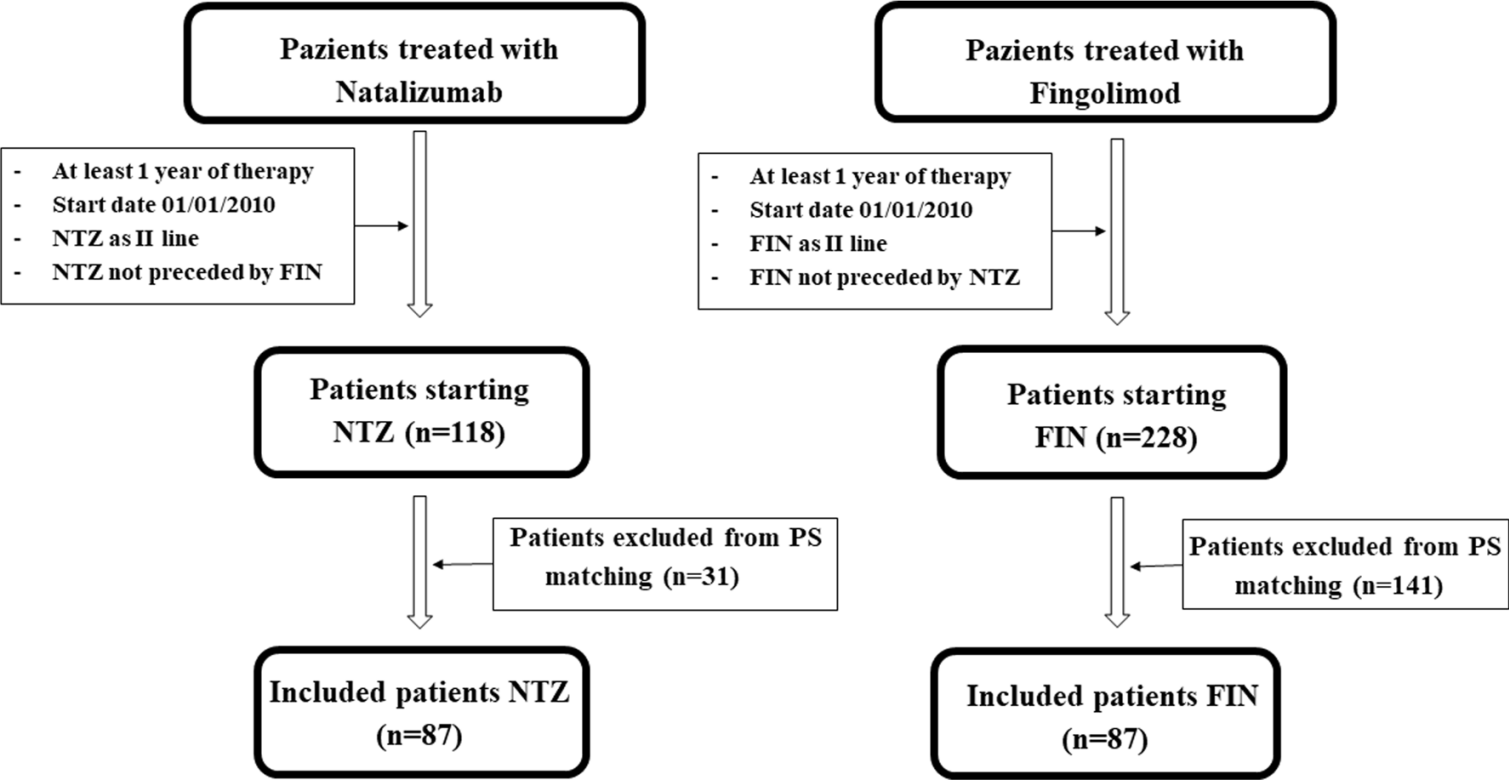
Patient disposition. Enrolment, inclusion, and matching of study population

**Fig. 2:**
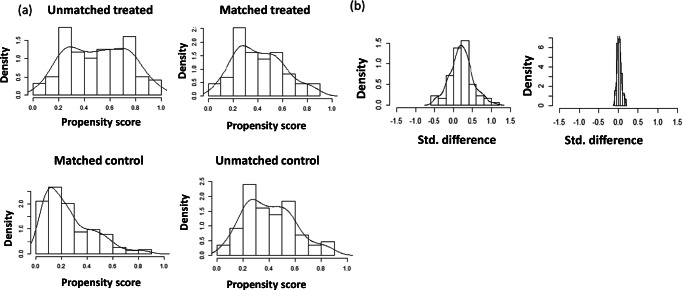
Propensity score (PS) matching performances: (**a**) PS distributions before and after pair-matching and (**b**) standardized mean difference (SMD) distribution of the entire matched cohort before and after pair-matching

At the end of March 2018, we identified 346 eligible patients switching from BRACE to either NTZ or FIN. Of these, 228 were treated with FIN, and 118 were treated with NTZ. During the time interval between 2011 and 2018 at our clinic, 37 patients stopped the NTZ treatment due to the concern of PML (they became positive to anti-JCV antibodies), and 9 discontinued NTZ due to pregnancy (programmed of confirmed) before reaching the 4 years of follow-up. The same apply to the fingolimod cohort, in which 7 patients did not complete the 4-year follow-up due to pregnancy (confirmed or planned) and 11 patients due to safety concerns (i.e., lymphocyte count reduction). Moreover, in the fingolimod cohort, 28 patients stopped the treatment due to lack of efficacy before the 4 years of follow-up with a mean (SD) time to treatment discontinuation of 2.73 ± 0.99 years. Patients’ demographic and clinical characteristics at baseline before and after the PS matching are shown in Table 1. The PS matching procedure retained 87 pairs of patients switching to NTZ (73.7%) or FIN (38.2%), respectively (Fig. 1). The matching procedure significantly improved the overall balance as indicated by the SMD before and after the procedures (Table 1). Moreover, the adequacy of the matching has been validated through graphic methods, deriving from the elaboration of the PS matching, namely, the PS graphs. The distribution of propensity score in the cohorts treated before and after the matching is portrayed in Fig. 2a. Figure 2b shows the SMD before and after the matching and the fifth chart the absolute standardized differences, namely, the RGraph.

**Table 1:** Baseline clinical and demographic characteristics before and after the PS matching

Variable	Natalizumab *N* = 118	Fingolimod *N* = 228	SMD	Natalizumab *N* = 87	Fingolimod *N* = 87	SMD
Sex (F/M)	79/39	146/82	0.061	57/30	56/31	2.096
Age at I infusion of the drug (mean ± SD) in years	34.86 ±11.86	37.81 ± 9.49	−27.455	36.72 ± 11.17	36.95 ± 9.07	−2.259
Disease duration (mean ±SD)	10.68 ± 7.49	11.89 ±7.52	−16.117	10.97 ± 6.87	11.08 ±7.41	−1.539
N. of previous total relapses (mean ±SD)	6.97 ±0.91	5.81 ±4.31	26.998	6.80 ± 4.06	6.28 ±4.81	1.168
N. of relapse previous year (mean ±SD)	1.39 ± 0.8	0.82 ± 0.7	73.19	1.17 ± 0.73	1.14 ± 0.82	3.830
Cumulative exposure period (mean ± SD) in years	4.81 ± 3.6	6.34 ± 4.39	−34.568	5.46 ± 3.73	5.31 ± 4.04	3.849
Wash out time (mean ± SD) in days	119.64 ± 228.13	95.74 ± 300	89.516	109.67 ± 212.84	94.24 ± 226.07	7.027
N. of patients with comorbidities, *n* (%)	75 (63.6%)	91 (39.9%)	0.492	51 (58.6%)	51 (58.6%)	0
N. of patients with new T2 lesions at MRIprior to treatment, *n* (%)	83 (70.3%)	119 (52.2%)	0.396	58 (66.7%)	43 (49.4%)	0.358
No. of new T2 lesions at MRI prior to treatment(mean ± SD)	1.76 ± 2.03	1.40 ± 2.2	16.710	1.52 ± 1.67	1.27 ± 1.87	1.634
N. of patients with Gd positive lesionsin T1 at the MRI prior to treatment, *n* (%)	58 (49.2%)	66 (28.9%)	0.406	35 (40.2%)	26 (29.9%)	0.210
No. of Gd-positive lesions in T1 at the MRIprior to treatment (mean ± SD)	1.13 ± 1.7	0.45 ± 0.9	47.909	0.75 ± 1.19	0.67 ± 1.41	6.109
EDSS (median; min–max)	3.75 (1.5–7.5)	3.0 (1–8)	−28.730	4.00 (1.5–7.5)	3.5 (1.5–8)	1.128

*ARR* annualized relapse rate, *Gd* gadolinium, *DMD* disease modifying drug, *SMD* standardized mean difference

In particular, before the PS matching, the two groups were unbalanced for many variables. Patients who escalated to NTZ were younger (mean ± SD, years: NTZ = 34.86 ± 11.86; FIN = 37.81 ± 9.49; SMD = −27.455), presented a higher number of relapses in the year prior the vertical switch (mean ± SD: NTZ = 1.39 ± 0.8; FIN = 0.82 ± 0.7; SMD = 73.19), a longer wash out period (mean ± SD, days: NTZ = 119.64 ± 228.13; FIN = 95.74 ± 300; SMD = 89.516), in comparison to those who received FIN. The number of new T2 lesions at MRI and of Gd-positive lesions in T1 prior to treatment was higher in the cohort of patient escalating to NTZ (mean ± SD: NTZ = 1.76 ± 2.03; FIN = 1.40 ± 2.2; SMD = 16.71; NTZ = 1.13 ± 1.7; FIN = 0.45 ± 0.9; SMD = 47.909, respectively) in comparison to those receiving FIN.

On the contrary, the cumulative exposure time to other DMTs (mean ± SD: NTZ = 4.81 ± 3.6; FIN= 6.34 ± 4.39; SMD = −34.568) and the number of patients with comorbidity (mean ± SD: NTZ = 75 (63.6%); FIN = 91 (39.9%); SMD = 0.492) were higher in patients receiving FIN in comparison to those who switched to NTZ.

The effect of treatment on disease activity was evaluated in 174 PS-matched RRMS patients receiving NTZ (*n*=87) or FIN (*n*=87).

At the second year of follow-up, in the FIN group, 82.7% of the patients were relapse free, 80.4% were free of new or enlarging T2 or Gd + lesions, and 77% were free of EDSS worsening. Therefore, 39 patients (44%) of the FIN group reached a NEDA-3 status at year 2. At the fourth year of follow-up, the figure was the following in the FIN group: 54% of the patients did not present relapses, 49.4% did not present new T2 or Gd + lesions, and 54 % did not present a worsening of EDSS. Therefore, 17 patients (25.8%) of the FIN group reached a NEDA-3 status at year 4.

Referring to the NTZ group, at the year 2 of follow-up, 87.3% of the patients were relapse free, 100% were free of new or enlarging T2 or Gd + lesions, and 96.5% were free of EDSS worsening. Therefore, 54 patients (63%) of the NTZ group reached a NEDA-3 status at year 2. At the fourth year of follow-up, the figure was the following: 77% of the patients did not presented relapses, 81.6% did not presented new T2 or Gd + lesions, and 78.1 % did not present a worsening of EDSS. Therefore, 32 patients (45.7%) of the NTZ group reached a NEDA-3 status at year 4.

**Fig. 3:**
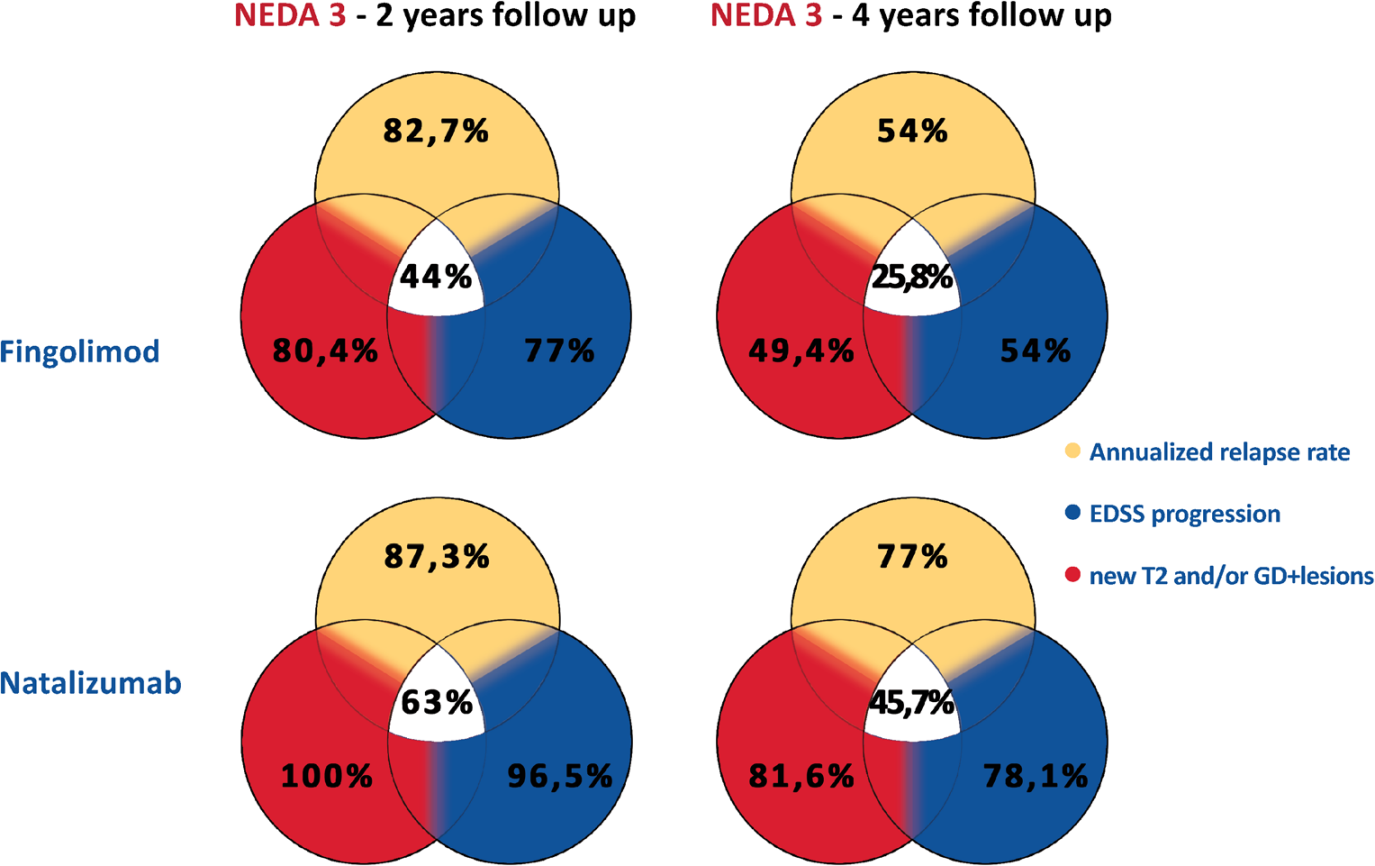
Primary and secondary outcomes (NEDA-3 and its components) investigated in propensity score-matched subsamples

Compared to FIN, the NTZ group presented a higher percentage of patients reaching the NEDA-3 status after 2 (63% (*n*=54) vs 44% (*n*=39), *p*=0.037) and 4 years of follow-up (45.7% (*n*=32) vs 25.8% (*n*=17), *p*=0.015) (Fig. 3).

**Fig. 4:**
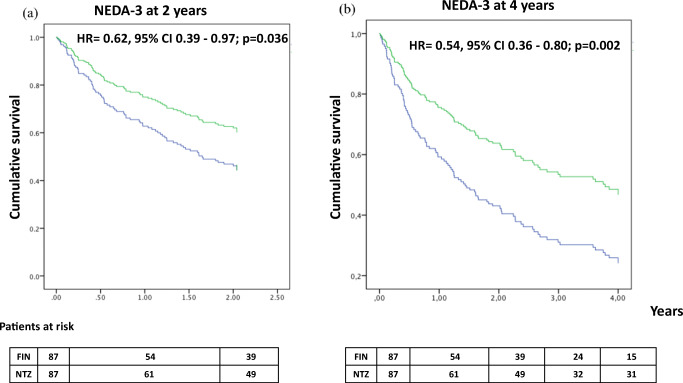
Risk of not achieving NEDA-3 status explored in matched samples by Cox proportional hazard regression models, adjusted for PS covariates, and stratified by matched cases at 2 years (**a**) and at 4 years (**b**)

The risk of not reaching the NEDA-3 status at 2 and at 4 years follow-up was significantly lower in patients receiving NTZ in comparison to patients treated with FIN (NEDA-3 at 2 years: HR= 0.62, 95% CI 0.39–0.97; *p*=0.036; NEDA-3 at 4 years: HR= 0.54, 95% CI 0.36–0.80; *p*=0.002) (Fig. 4).

Finally, we evaluated the number of patients who presented an EDSS worsening in the absence of clinical or MRI activity. At the 2-year follow-up, 13 patients reported only a worsened EDSS score: 10 patients in the group treated with FIN (8.7%) and 3 patients (2.6 %) treated with NTZ. At the 4-year follow-up, only 2 patients reported an EDSS worsening without clinical or MRI activity, one for each treatment group.

## Discussion

The results of this study confirm that NTZ is more effective than FIN in patients who do not respond to first-line agents, as already suggested in previous observational studies. In clinical practice, NTZ and FIN have been both used as the first option in patients with rapidly worsening MS or in patients who do not respond to first-line DMTs. There are no specific criteria to guide the choice between the two treatments, which still remain empirical and entrusted to clinical intuition [18]. Moreover, MS experts have to carefully evaluate the benefit/risk balance related to DMTs, mainly considering the risk of progressive multifocal leukoencephalopathy in John Cunningham-virus seropositive patients associated to the exposure to NTZ and infective, cardiac, ocular, and dermatologic complications in those treated with FTY [27–33].

Evidence also suggests that achieving NEDA-3 improves prognosis in RRMS [17]. However, NEDA-3 is not included in current guidelines as a treatment target, even though treating-to-target strategies have been used in other chronic autoimmune disorders [24]. The treatment of MS is complicated by variability in the disease course and the often-uncertain relation between disability worsening, relapses, and long-term outcomes.

Several observational studies showed the superiority of NTZ compared to FIN in RRMS patients non-responders to first-line therapy. The efficacy of NTZ is underlined by an observational study in which, compared with FIN, the NTZ group presented a higher proportion of relapse-free patients and a higher proportion of patients without evidence of pathological activity after 2 years of follow-up, although the worsening of disability was not statistically different in the two groups [13]. The proportion of patients reaching NEDA-3 is pointed out as main outcome measure in another study [17] and provides real-world evidence that NTZ can be considered more effective than both FIN and self-injectable drugs in non-responders. Similar findings can be found in an Italian observational, retrospective study, in which after 24 months, although both drugs resulted highly effective, in the NTZ group was observed a lower relapse risk and higher time to first relapse, a lower MRI activity, and a higher proportion of patients with confirmed regression of disability and 2-year NEDA [14].

In another study from the MSBase international database, patients who experienced relapse or progression of disability were identified within 6 months immediately prior to switching to NTZ or FIN [20]. The outcomes examined demonstrated that in the active phase of the pathology, the transition to NTZ can be considered more effective than the transition to FIN in reducing the worsening of disability in the short term.

The findings of a Danish study using data from a large cohort of RRMS patients in the Danish Multiple Sclerosis Treatment Register offer a different point of view, as they found no substantial differences in clinical activity between NTZ- and FIN-treated patients [34].

Our results, obtained by studying the 87 pairs generated by the PS matching procedure, confirm and extend the results of previous observational studies, by studying for the first time the comparative effectiveness of NTZ and FIN up to 4 years.

We found a drastic reduction in the number of relapses during both treatments. The ARR was still higher in the first year of treatment, a sign of the residual but still powerful inflammatory activity of the pathology, but then it decreases until it remains constant in the following years.

Radiological evidences are often not considered in many observational studies, because of either the difficult availability of data or the subjectivity of reporting or the absence of a precise count of the lesions [21]. In this study, MRI findings offered an interesting insight into the effectiveness of the therapies: compared to baseline, we find a reduction in the percentage of patients with an increase in the number of lesions or with an increased volume of the same or with active lesions in both cohorts.

The percentages of patients maintaining the NEDA-3 status at year 2 in our cohort (NTZ 63%, FIN 44%) were in line with previous reported values (Prosperini et al. NTZ 67%, FIN 42%; Baroncini et al. NTZ 70%, FIN 44%) [13, 17].

Moreover, we have studied patients continuously exposed to NTZ or FIN up to 4 years, confirming at year 4 the superiority of NTZ in terms of achieving NEDA-3 status.

In our cohort of patients exposed to second-line agents, no serious adverse events (SAE), defined according to European Medicines Agency (EMA) guidelines [31] [32], occurred during the follow-up.

Some limitations of this study deserve discussion. First, although we have applied the PS matching analysis to mitigate the known treatment indication bias, the lack of randomization and blinded evaluation of outcomes remain implicit limits common to all observational studies.

Second, as in all the previous similar studies which have addressed the same topic, the original cohorts were mainly imbalanced because of the predominance of more “active” patients in the NTZ group [13, 14, 16, 20, 22].

In addition, the matching procedure resulted in the exclusion of the less active patients in the FIN group and more active patients in the NTZ group. We evaluated the baseline clinical and demographic characteristics of patients excluded from PS matching, comparing them with those of the cohorts of matched patients (Table 2). The analysis showed that patients treated with NTZ excluded from matching had a more aggressive form of disease in terms of clinical and radiological activity. On the contrary, the unmatched FIN cohort had a less clinically and radiologically active disease. This type of selective exclusion might have favored the FIN group in the comparison, thus reducing our ability to show the real impact of NTZ treatment on the NEDA-3 status.

**Table 2:** Baseline clinical and demographic characteristics of the PS matching excluded and included cohorts

Variable	Natalizumab *N* = 31	Fingolimod *N* = 141	Natalizumab *N* = 87	Fingolimod *N* = 87
Sex (F/M)	22/9	90/51	57/30	56/31
Age at I infusion of the drug (mean ± SD) in years	31.41 ± 12.85	39.19 ± 9.66	36.72 ± 11.17	36.95 ± 9.07
Disease duration (mean ±SD)	9.87 ± 9.09	12.39 ± 7.57	10.97 ± 6.87	11.08 ±7.41
N. of previous total relapses (mean ±SD)	7.45 ± 4.96	5.51 ± 3.97	6.80 ± 4.06	6.28 ±4.81
N. of relapse previous year (mean ±SD)	2.03 ± 0.83	0.61 ± 0.56	1.17 ± 0.73	1.14 ± 0.82
Cumulative exposure period (mean ± SD) in years	2.96 ± 2.73	6.97 ± 4.48	5.46 ± 3.73	5.31 ± 4.04
Wash out time (mean ± SD) in days	147.61 ± 268.35	96.67 ± 339.78	109.67 ± 212.84	94.24 ± 226.07
N. of patients with comorbidities, *n* (%)	24 (77.4%)	40 (28.4%)	51 (58.6%)	51 (58.6%)
N. of patients with new T2 lesions at MRI prior to treatment, *n* (%)	25 (80.6%)	76 (53.9%)	58 (66.7%)	43 (49.4%)
No. of new T2 lesions at MRI prior to treatment (mean ± SD)	2.41 ± 2.72	0.61 ± 1.22	1.52 ± 1.67	1.27 ± 1.87
N. of patients with Gd-positive lesions in T1 at the MRI prior to treatment, *n* (%)	23 (74.2%)	40 (28.4%)	35 (40.2%)	26 (29.9%)
No. of Gd-positive lesions in T1 at the MRI prior to treatment (mean ± SD)	2.19 ± 2.49	0.31 ± 0.56	0.75 ± 1,19	0.67 ± 1.41
EDSS (median; min–max)	3.90 (1.5–7.0)	3.19 (1–6.5)	4.00 (1.5–7.5)	3.5 (1.5–8)

*ARR* annualized relapse rate, *Gd* gadolinium, *DMD* disease modifying drug, *SMD* standardized mean difference

Although this is a single-center study, MRI acquisition was made in different radiological centers using a non-standardized protocol.

Finally, strength and limitations of using NEDA as study outcome have to be considered. NEDA is a composite score able to combine different aspects of MS disease activity. However, the imbalance between the different component measures, the dominant role of MRI activity in determining loss of NEDA, and no standardized definitions of this composite score still remain significant limits [35].

In conclusion, our results suggest that both NTZ and FIN are highly effective in reducing relapse risk, MRI, and EDSS worsening. However, NTZ is more effective than FIN in achieving a NEDA-3 status in patients with a highly active disease not controlled by BRACE. The concept of NEDA is on the way of being increasingly considered as a basic parameter to be pursued in studies of effectiveness and efficacy in the era of DMTs. However, further work is required to clarify the valence of the NEDA status as long-term outcomes and its sustainability in real-life setting.
